# Hydrocele of the Canal of Nuck: A Rare Case

**DOI:** 10.7759/cureus.63672

**Published:** 2024-07-02

**Authors:** Divya Lakshmi A, Balaji Subramaniam

**Affiliations:** 1 Department of Pediatrics, Sree Balaji Medical College & Hospital, Chennai, IND; 2 Department of Urology, SRM Medical College Hospital & Research Centre, Chennai, IND

**Keywords:** pelvic mri, imaging, excision, inguinal, hydrocoele

## Abstract

Hydrocele of the canal of Nuck in adults is an extremely rare condition. It results from the incomplete obliteration of the processus vaginalis, which is a tubular structure that connects the peritoneal cavity to the labia majora during foetal development. Failure of this structure to close can lead to the accumulation of peritoneal fluid in the inguinal canal, resulting in a hydrocele. A 40-year-old lady presented to our OPD with a history of left groin pain and tenderness of one-year duration. No obvious swelling was noted clinically. No other clinical abnormality was seen. An ultrasonogram and MRI of the pelvis played a key role in arriving at a diagnosis. She was then taken up for excision of the hydrocele along its entire extent. Though a rare presentation, it should be among the differential diagnosis in a female with inguinal pain or an inguinal swelling. Accurate diagnosis through imaging and appropriate surgical management can lead to favourable outcomes.

## Introduction

The inguinal canal houses different structures in males and females, leading to distinct clinical considerations. In males, the spermatic cord travels through the canal, while in females, it contains the round ligament of the uterus. The round ligament originates from the cornu of the uterus, near the fallopian tube, and extends through the inguinal canal to attach to the labia majora. Within the canal, the round ligament is enveloped by a small peritoneal pouch known as the ‘canal of Nuck’ [1]. It is the female equivalent of the processus vaginalis in males, which normally obliterates. When there is a failure of complete obliteration, it can lead to an indirect type of inguinal hernia as well as ‘hydrocele of the canal of Nuck’. This is of interest because of the diagnostic difficulty involved, especially when the patient does not present with an obvious inguinal swelling.

## Case presentation

A 40-year-old lady presented with pain in the left groin of one-year duration with no obvious inguinal swelling. It was initially intermittent, after which she developed continuous, colicky, non-radiating pain that was not relieved with any medications. There was no history of abdominal pain, distension, vomiting, trauma, or fever. There were no disturbances in her bowel or bladder habits. A physical examination did not reveal any abnormality in the bilateral inguinal region, with no visible cough impulse. She had mild tenderness in the medial half of the left inguinal region. External genitalia and other systems were normal.

**Figure 1:**
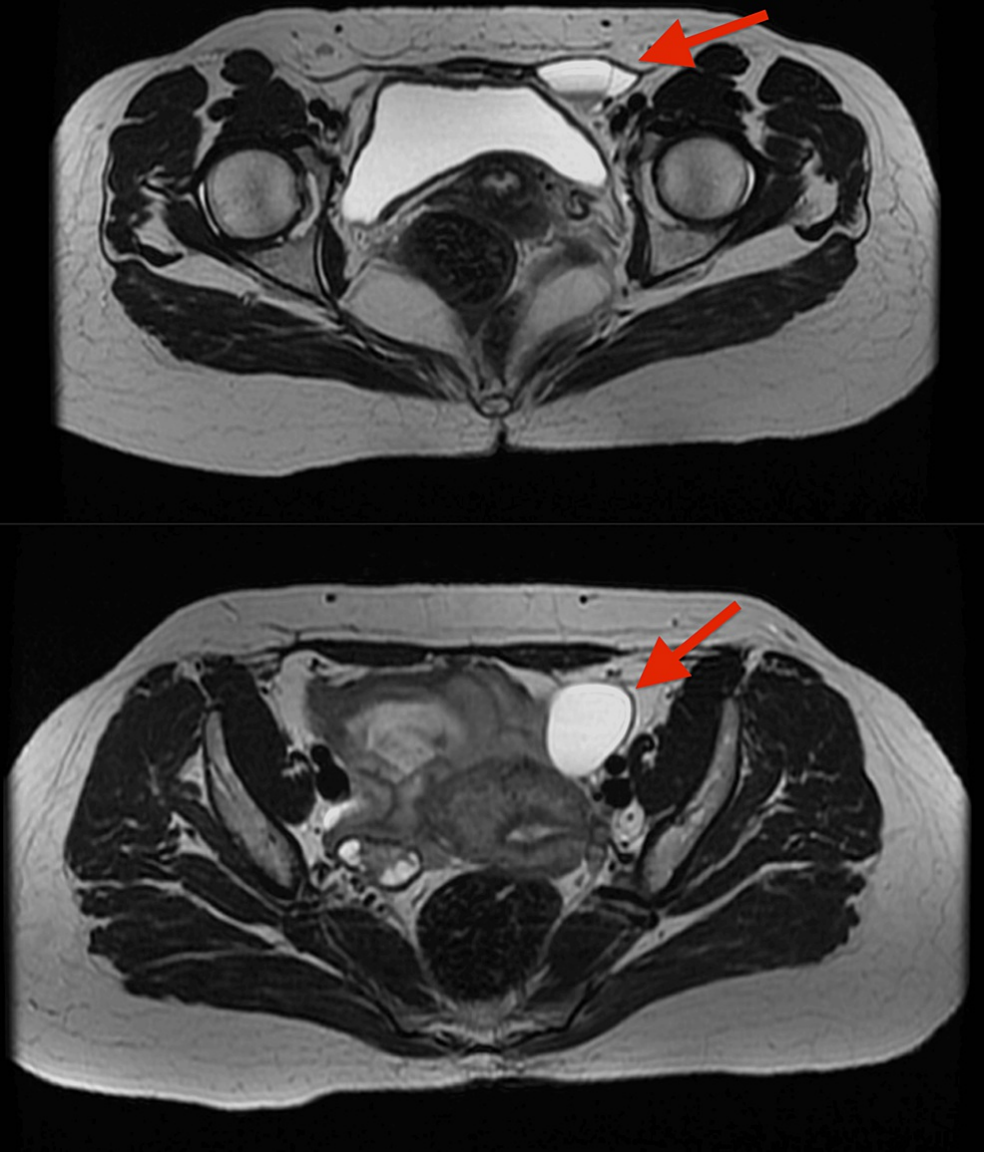
MRI imaging of the pelvis demonstrating a well-defined T2 hyperintense cystic lesion in the pelvis with extension into the inguinal canal (top) with intraperitoneal extension leading to the uterus (bottom)

An ultrasonogram of the abdomen showed a 4x1 cm linear anechoic collection in the left inguinal region with few internal septations. To correlate, pelvis MRI was done, which revealed a well-defined T2 hyperintense cystic lesion in the pelvis with extension into the inguinal canal along the round ligament measuring 3.2x3.7x6.5 cm, giving an impression of hydrocele of the canal of Nuck (Figure 1). All routine pre-operative parameters were normal.

**Figure 2:**
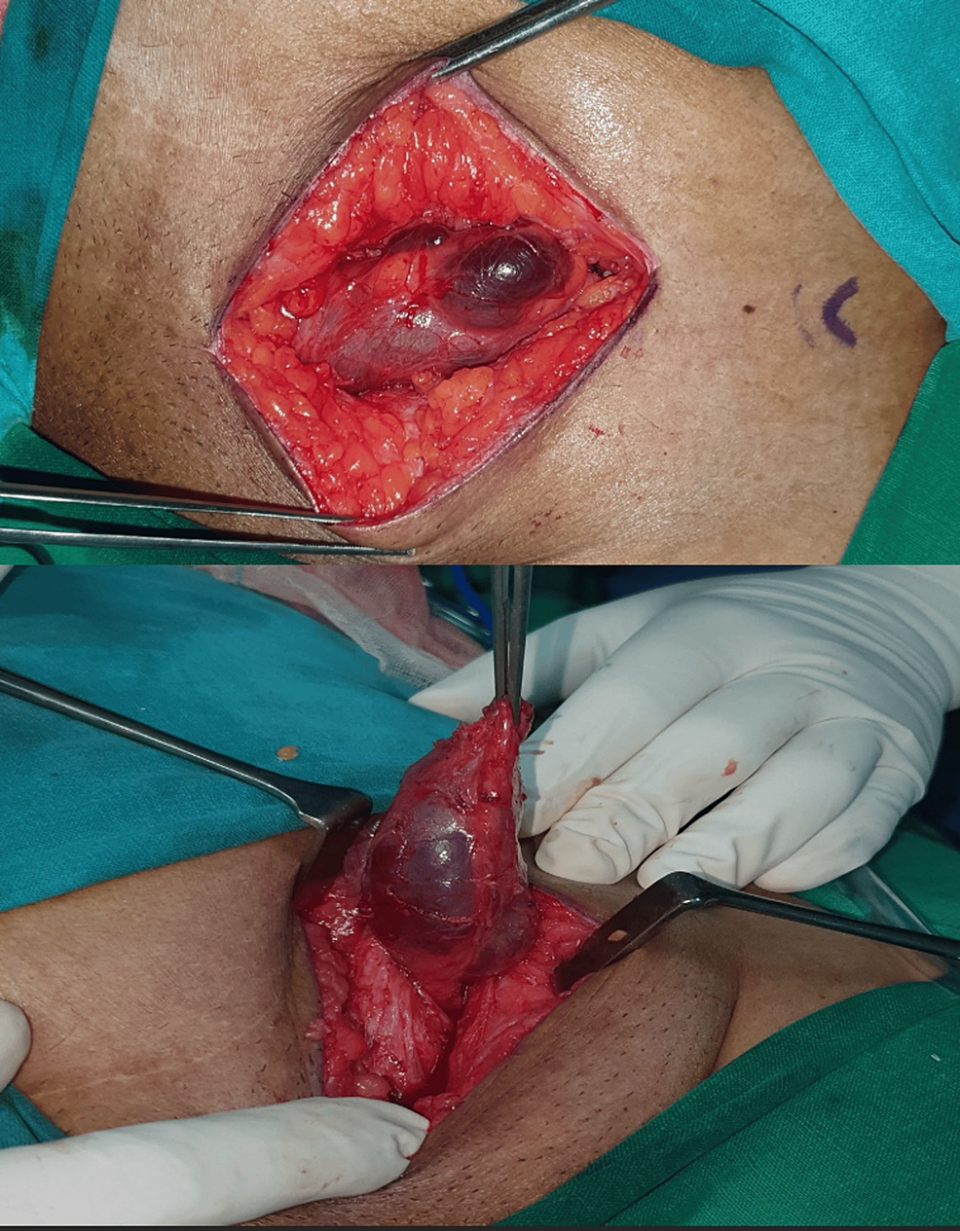
Intraoperative pictures showing the intact hydrocele of the canal of Nuck in the inguinal canal

Hence, after obtaining consent, she underwent surgical exploration of the left inguinal region under spinal anaesthesia. In the inguinal canal, an hourglass-shaped hydrocele of the canal of Nuck with the lateral end extending beyond the peritoneum was found. It was traced till the deep ring and the peritoneal cavity was opened (Figure 2). The lateral end of the hydrocele sac was traced along the round ligament till the body of the uterus. The excess sac was excised in toto along with the round ligament till the uterus. The defect was closed in layers along with posterior wall strengthening by a modified Bassini repair. The patient had an uneventful postoperative stay and was discharged by postoperative day 2.

## Discussion

When the female foetus undergoes intrauterine development, the round ligament of the uterus descends through the inguinal canal to the labia majora. The round ligament may drag along a part of the peritoneal fold. This is called the 'canal of Nuck'. This was first described in 1691 by Anton Nuck, a Dutch anatomist. The peritoneal fold usually gets obliterated during early infancy, failing which, it may lead to the development of an indirect inguinal hernia or a hydrocele.

In real-world statistics, a congenital hydrocele or hernia is much more commonly seen in male children than in female [2]. Enlargement of the cyst with associated tenderness may be due to an increase in the secretion of fluid by the mesothelial cell lining of the peritoneal fold or due to its decreased absorption. Although it is idiopathic in most cases, some cases may be due to impaired lymphatic drainage, trauma, or inflammation.

Hydrocele of the canal of Nuck may be easily mistaken for an inguinal hernia because of the similar presentation; at least one third of the cases are also associated with an inguinal hernia [3]. Clinically, the patient presents with a painless or mildly painful, translucent, irreducible lump in the groin, specifically the inguino-labial region. But the overlying fascia and thick external oblique muscle and aponeurosis may not allow transillumination.

When clinical diagnosis is challenging, imaging studies may help with the pre-operative diagnosis, but most cases of the hydrocele of the canal of Nuck are diagnosed on the operating table during surgical exploration. Radiological diagnosis may be done by an ultrasonogram or MRI scan. The sonography findings usually show an anechoic or hypoechoic superficial mass lying in the groin, medial to the pubic bone at the level of the superficial inguinal ring with no peripheral vascularity. MRI findings include a well-defined, thin-walled cystic lesion, hypointense on T1 and hyperintense on T2 weighted images [4]. Surgery is necessary for the final diagnosis and treatment. Considering the common association with indirect inguinal hernias, dissection must be done up to the deep inguinal ring, along with ligation of the neck of the peritoneal pouch. This can also be managed by a laparoscopic approach [5].

## Conclusions

While the patients commonly present with an inguinal lump resembling a hernia, our patient presented with inguinodynia with no obvious swelling or cough impulse. Among the wide spectrum of differential diagnosis available, a keen eye of suspicion helped in the early diagnosis of the condition and adequate surgical planning.
